# Quercetin and quercetin 3-*O*-glycosides from *Bauhinia longifolia* (Bong.) Steud. show anti-Mayaro virus activity

**DOI:** 10.1186/1756-3305-7-130

**Published:** 2014-03-28

**Authors:** Alda E dos Santos, Ricardo M Kuster, Kristie A Yamamoto, Tiago S Salles, Renata Campos, Marcelo DF de Meneses, Márcia R Soares, Davis Ferreira

**Affiliations:** 1 Natural Product Research Institute, Center of Health Sciences, Federal University of Rio de Janeiro, Rio de Janeiro, Brazil; 2 Microbiology Institute, Virology Department, Federal University of Rio de Janeiro, Rio de Janeiro, Brazil; 3 Chemistry Institute, Biochemistry Department, Federal University of Rio de Janeiro, Rio de Janeiro, Brazil; 4 National Institute of Science and Technology for Structural Biology and Bioimaging, Rio de Janeiro, Brazil

**Keywords:** *Bauhinia longifolia*, Flavonoids, Quercetin derivatives, Antiviral, MAYV

## Abstract

**Background:**

The arthropod-borne Mayaro virus (MAYV) causes ‘Mayaro fever’, a disease of medical significance, primarily affecting individuals in permanent contact with forested areas in tropical South America. Recently, MAYV has attracted attention due to its likely urbanization. Currently, there are no licensed drugs against most mosquito-transmitted viruses. Here, we investigated the *in vitro* anti-MAYV activity of the flavonoids quercetin and its derivatives from the Brazilian shrub *Bauhinia longifolia* (Bong.) Steud.

**Methods:**

Flavonoids were purified by chromatographic fractionation from leaf extracts of *B. longifolia* and chemically identified as quercetin and quercetin glycosides using spectroscopic techniques. Cytotoxicity of purified flavonoids and of EtOAc- and *n*-BuOH-containing flavonoid mixtures was measured by the dye-uptake assay while their antiviral activity was evaluated by a virus yield inhibition assay.

**Results:**

The following flavonoids were purified from *B. longifolia* leaves: non-glycosylated quercetin and its glycosides guaijaverin, quercitrin, isoquercitrin, and hyperin. EtOAc and *n*-BuOH fractions containing these flavonoids demonstrated the highest antiviral activity of all tested substances, while quercetin had the highest antiviral activity amongst purified flavonoids. Quercetin, EtOAc, or *n*-BuOH fractions inhibited MAYV production by more than 90% at 25 μg/mL, displaying a stronger antiviral effect than the licensed antiviral ribavirin. A mixture of the isomers isoquercitrin and hyperin had a modest antiviral effect (IC_90_ = 104.9), while guaijaverin and quercitrin did not show significant antiviral activity.

**Conclusions:**

*B. longifolia* is a good source of flavonoids with anti-Mayaro virus activity. This is the first report of the activity of quercetin and its derivatives against an alphavirus.

## Background

Brazil remains extensively covered by tropical forests and other natural ecosystems, despite high deforestation rates. A critical effect of deforestation is the introduction of new viruses into urban areas, providing ideal conditions for the development of several arboviruses [1]. Indeed, more than 200 species of arboviruses have been isolated in urban areas in Brazil, including approximately 40 species causing diseases in humans, including the Mayaro fever [2], which is caused by the Mayaro virus (MAYV).

Mayaro virus (MAYV), a member of the Togaviridae family (genus Alphavirus), was first isolated in Trinidad in 1954 [3]. MAYV is closely related to Chikungunya and other alphaviruses, producing an acute, self-limited dengue-like illness that is accompanied by long-lasting arthralgia [4,5]. Since the initial description of MAYV, several small outbreaks of Mayaro fever have been reported in rural communities in northern Brazil and in eastern Bolivia [6-8]. Moreover, MAYV antibodies have been detected in human and animal populations in Bolivia, Brazil, Colombia, Panama, Peru, Surinam, Trinidad and Tobago, Venezuela, French Guiana, and Mexico, suggesting increasing viral dissemination [9-15].

Antibodies against MAYV are often detected in certain species of monkeys, which are likely to be the main natural reservoir of the virus [16], while the main vectors are mosquitoes of the *Haemagogus* species, although *Aedes* species might also act as MAYV vectors. Thus, it is possible that, under appropriate conditions, MAYV can be transmitted in urban areas [1].

In Brazil, MAYV is endemic to the Amazon region, but cases of the disease have been observed elsewhere. Mayaro fever outbreaks occurred in central regions in Brazil, namely Itarumã (Goiás state) and in Peixe (Tocantins state) [17], in 1987 and 1991, respectively. Also, three cases of Mayaro fever were reported in Mato Grosso state [18]. Also, a report from 2010 describes a young French tourist being diagnosed with MAYV after visiting the Brazilian Amazon [19]. The importance of controlling MAYV spread or seeking treatment for the virus infection extends beyond the Amazon region. As an alphavirus, MAYV can serve as a model for other important viruses such as Chickungunya virus, an emerging virus that has been spread in Europe recently [20-22].

Currently, about 40 antiviral drugs are available for clinical use, mainly targeting HIV and a small number of other viruses [23]. Nevertheless, research efforts to explore the potential of natural products as sources of novel low toxicity and high selectivity antiviral substances have increased lately [24]. These natural products, also called plant-derived products, are very attractive when compared to synthetic molecules. This is true mainly because of the low cytotoxicity, the rapid degradation in the environment, and because of the complexity of the chemistry in these products, that should limit resistance and increase the applicability of use, such as vector control studies [25,26]. Because there are many approaches for the use of natural products, the modes of action or the active components they contain and the metabolic pathways they interact with must be studied. This can be accomplished initially by *in vitro* studies such as the cell culture approach in this paper.

Shrubs from the *Bauhinia* (Fabaceae) genus are found in tropical regions of Asia, Africa, and Central and South America. In Brazil, these plants are known as ‘pata-de-vaca’ (cow’s foot) because of their leaf shape [27]. Tea made from *B. longifolia* and *B. forficata* leaves is consumed in certain regions of Brazil [28] for its anti-diabetic properties (according to Brazilian folk medicine). Flavonoids, mainly kaempferol and quercetin derivatives, are commonly found in plants in the *Bauhinia* genus [29]. Flavonoids have known antiviral potential, and a number of reports describe their different antiviral mechanisms, including inhibition of infectivity and replication, depending on the target virus [30].

In view of this interesting scenario, in the present study we evaluated the antiviral activity of *Bauhinia longifolia* (Bong.) Steud. against MAYV replication in Vero cells. We also determined the selective antiviral activity of purified quercetin and quercetin glycoside derivatives, as well as of leaf extracts rich in these flavonoids.

## Methods

### Plants, cells and viruses

In this study, leaves from wild specimens of *Bauhinia longifolia* (Bong.) Steud. collected in the municipality of Luz, Minas Gerais state (Brazil) were used. Plant species authentication was performed by comparison with herbarium specimens from the Institute of Biological Sciences (Federal University of Minas Gerais, Belo Horizonte, Brazil), where a voucher specimen (BHCB 18778) was deposited.

Vero cells (African green monkey kidney, ATCC CCL-81) were grown at 37°C with 5% CO_2_, in Dulbecco’s modified Eagle’s medium (DMEM) (Life Technologies, USA) supplemented with 5% fetal bovine serum (Cultilab, BRA), 50 IU/mL of penicillin, and 50 μg/mL of streptomycin (Sigma-Aldrich, USA). Mayaro viruses (ATCC VR-66, lineage TR 4675) were propagated in Vero cells and viral stocks in 10% glycerol were kept at -70°C. Virus titer was determined by plaque assay (described under ‘Antiviral activity assay’).

### Extraction, fractionation, and purification of quercetin derivatives

Air-dried and powdered leaves (1.5 Kg) were extracted with MeOH at room temperature for 7 days. Pooled methanol extracts were filtered and concentrated under reduced pressure to produce a crude extract (95 g), which was diluted in MeOH-H_2_O to a ratio of 9:1, and then extracted successively with *n*-hexane, CH_2_Cl_2_, EtOAc, and *n*-BuOH.

EtOAc (7 g) and *n*-BuOH (6 g) fractions were chromatographed separately on amberlite XAD-16 columns (2 m × 8 cm i.d.) (Sigma-Aldrich). Aqueous methanol solutions (from 0% to 100%, with 10% increments) were used as the mobile phase, and 11 fractions were collected for each extract (fractions A1–A11 and B1–B11, for EtOAc and *n*-BuOH, respectively). After thin layer chromatography (TLC) analysis, selected fractions were pooled in three groups: F1, including fractions A6–A9 (4.5 g); F2, including fractions A10–A11 (0.9); and F3, including fractions B6–B11 (1.9).

F1 and F3 were chromatographed separately on Sephadex LH-20 columns (30 cm × 45 mm) (Sigma-Aldrich) with MeOH-H_2_O (1:1) as the mobile phase. F1 chromatography yielded fractions containing guaijaverin (**1**) and quercitrin (**3**), F2 fractions contained aglycon quercetin (**2**), and F3 fractions contained a mixture of the isomers isoquercitrin (**4a**) and hyperin (**4b**).

### Reverse-phase HPLC-DAD and TLC analyses

HPLC-DAD (High Performance Liquid Chromatography with Diode Array Detector) and TLC (Thin Layer Chromatography) were used to analyze the chemical composition of EtOAc and *n*-BuOH fractions described above. The mobile phase of HPLC-DAD analysis consisted of (A) 1% phosphoric acid in water or (B) 1% phosphoric acid in methanol. Gradients used were: 0–15 min 50–70% of B followed by 15–25 min 70–100% of B. The flow rate was 1 mL/min and the injection volume 20 μL. The UV–vis spectra were recorded from 254 to 400 nm, with detection at 254 and 365 nm. TLC was performed on silica gel Plates 60 F_254_ (Merck, 20 × 20 cm, 0.5 mm thickness), using ethyl acetate-methanol–water-acetic acid (8:1:0.5:0.5) as eluent. After elution, TLC plates were observed under 254 nm UV light and then sprayed with solutions of NP (2-aminoethyldiphenylborinate 1% in methanol) and PEG-400 (polyethylene glycol 5% in ethanol) (both by Sigma-Aldrich, USA) before examination under 365 nm UV light. The presence of sugars was confirmed by acid hydrolysis of glycosides on TLC plates, and the sugar composition was inferred by comparison with standards of each of the following sugars: arabinose, xylose, galactose, glucose, and rhamnose (Sigma-Aldrich, USA). Final compound identification was performed by extensive 1D- (^1^H and ^13^C) and 2D- (COSY, HMBC, HSQC) NMR experiments, ESI-MS, UV spectral analysis, and by comparison with literature values.

### Cytotoxicity and antiviral activity assays

Cytotoxicity analysis of *B. longifolia*-derived flavonoids, EtOAc and *n*-BuOH fractions, was performed using a dye-uptake method modified from Borenfreund and Puerner [31]. Briefly, confluent cultures of Vero cells in 96-well microplates were treated with culture media containing different concentrations of the substances being tested. After 24 hours, the culture medium was replaced by a solution of 50 μg/mL neutral red and the cells were incubated for 3 hours, at 37°C, 5% CO2. Cells were then fixed with 20% formaldehyde and extracted with 50% methanol and 1% acetic acid, before measurement of absorbance at λ490 nm, using a spectrophotometer, to detect neutral red incorporation by living cells. Absorbance results were used to calculate, by regression analysis, the concentrations of the tested substances capable of reducing cell viability by 50% and 90% relative to controls (CC_50_ and CC_90_, respectively).

Antiviral activity was evaluated using a virus yield inhibition assay. Briefly, confluent cell monolayers grown in 24-well plates were infected with MAYV (multiplicity of infection = 0.1) for 1 hour, then rinsed with PBS and treated for 24 hours (at 37°C and 5% CO_2_) with different concentrations (0–100 μg/ml) of flavonoids or extracts diluted in culture medium. After treatment, culture supernatants were recovered and used for titration of extracellular infectious virus particles. For virus titration, confluent cell monolayers in 24-well plates were infected with serial dilutions of recovered supernatants for 1 hour at 37°C, 5% CO2. After inoculum removal, cells were rinsed with PBS and the monolayer was incubated with fresh medium with 2% carboxymethylcellulose (Sigma-Aldrich, USA) for 48 hours at 37°C, 5% CO2. Finally, cells were fixed with 20% formaldehyde and stained with 0.5% crystal violet in 20% ethanol, and viral plaques were counted. Ribavirin (Sigma-Aldrich, USA) was used as positive control for MAYV replication inhibition. For each substance or extract, IC_50_ and IC_90_ values were calculated and used to obtain a selectivity index (SI), expressed as the ratio CC_50_/IC_50_, and to estimate relative potency (RP) as the ratio between ribavirin (reference substance) IC_90_ and the tested substance’s IC_90_. Results were presented as mean inhibitory/cytotoxic concentration ± SD, and *t*-tests were used to evaluate the statistical significance of treatments relative to controls. P-values < 0.05 were considered statistically significant.

## Results

### Flavonoids quercetin and quercetin derivatives are found in extracts of *B. longifolia* leaves

**Figure 1:**
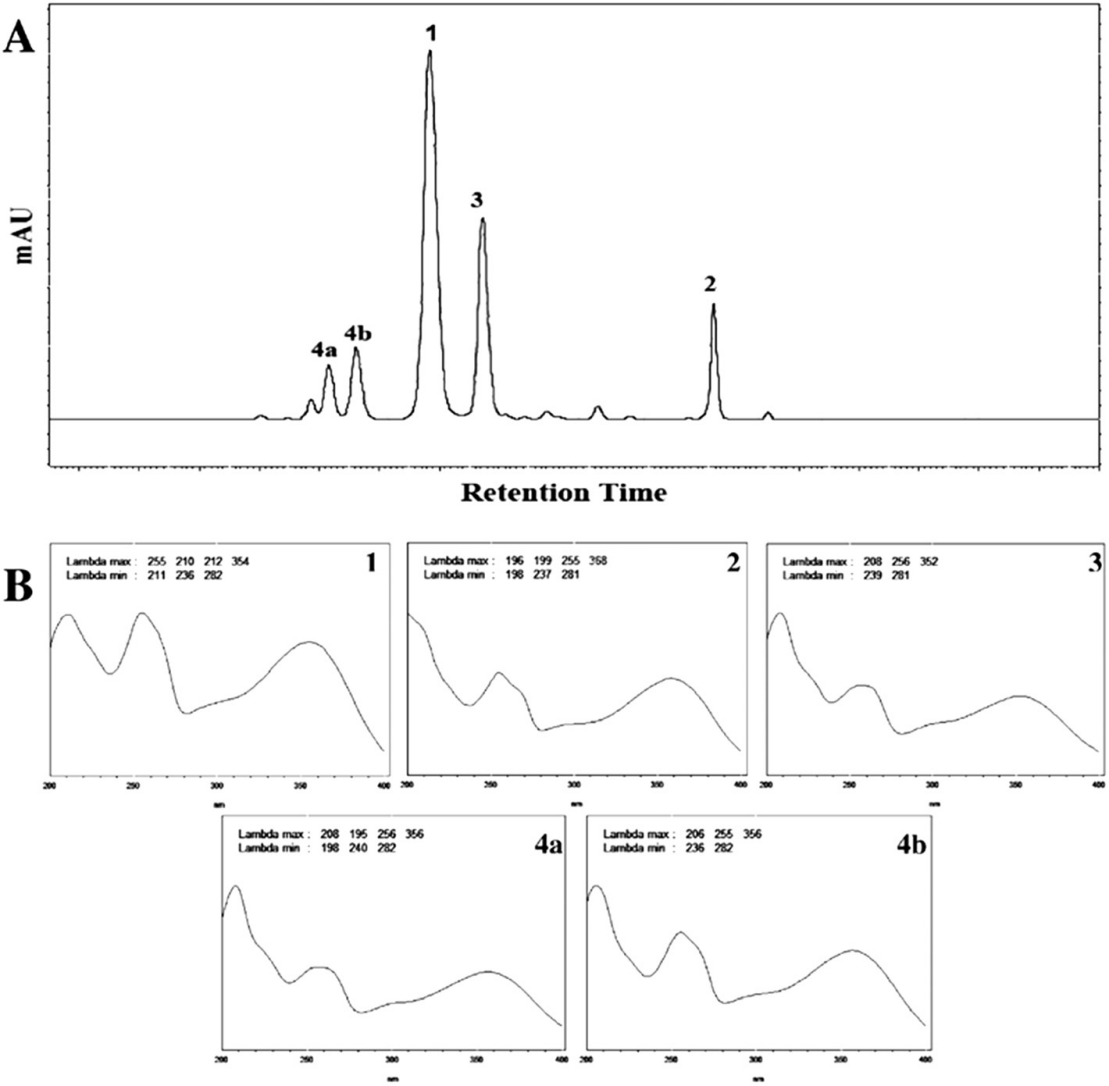
**HPLC-DAD analysis detects flavonoids in extracts from *B. longifolia*. (A)** 365 nm diode array chromatogram of EtOAc fraction from the leaves of *B. longifolia*. **(B)** UV spectra of quercetin derivatives present in the EtOAc fraction.

HPLC-DAD analysis revealed that EtOAc and *n*-BuOH fractions from the methanol extract of *B. longifolia* show similar chemical profiles (Figure 1A), displaying varying proportions of five major compounds having UV absorption typical of flavonoids (Figure 1B).

**Figure 2:**
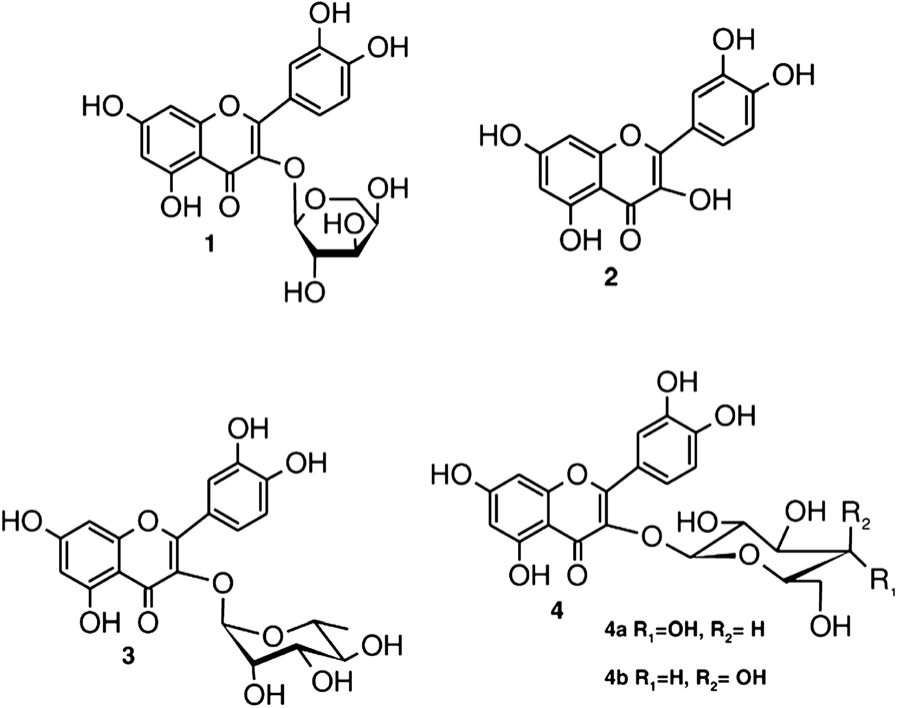
**Chemical structures of flavonoids identified in *B. longifolia*.** (1) guaijaverin (quercetin-3-*O*-α-arabinoside); (2) quercetin; (3) quercitrin (quercetin-3-*O*-α-rhamnoside); (4a) isoquercitrin (quercetin-3-*O*-β-glucoside); and (4b) hyperin (quercetin-3-*O*-β-galactoside).

Using NMR, ESI-MS, and UV spectral analyses, the compounds in profile peaks from Figure 1A were identified as: guaijaverin (quercetin-3-*O*-α-arabinoside, peak **1**) [32]; quercetin (peak **2**) [33]; quercitrin (quercetin-3-*O*-α-rhamnoside, peak **3**) [34]; isoquercitrin (quercetin-3-*O*-β-glucoside, peak **4a**); and hyperin (quercetin-3-*O*-galactoside, peak **4b**) [35]. Figure 2 shows the chemical structure of these compounds, and Table 1 shows their corresponding HPLC-DAD and ESI-MS data.

**Table 1:** **HPLC-DAD and ESI-MS data for quercetin derivatives from the leaves of *B*. *longifolia***

		***t***_**R**_	**[M-H]**	**λ **_**max**_
**Compound**	**Name**	**(min)**	**( *m/z *)**	**(nm)**
1	Guaijaverin	12.7	433	255, 354
2	Quercetin	22.1	301	255, 368
3	Quercitrin	14.4	447	256, 352
4a	Isoquercitrin	9.3	463	256, 356
4b	Hyperin	10.2	463	255, 356

^*tR*^ – retention time (in min).

^[M-H]-^ – pseudo molecular ion observed on the ESI-MS spectra at the negative mode.

^λ max^ – maximum absorption bands in the UV spectra.

The simplicity of the foliar flavonoid metabolism of *B. longifolia* is revealed by the absence of kaempferol derivatives (commonly found in the genus) and by the identification of quercetin derivatives only. Furthermore, the presence of quercetin 3-*O*-monoglycosides suggests the effective action of glycosilation enzymes such as 3-*O*-transferases [36].

### Cytotoxicity and antiviral activity

**Figure 3:**
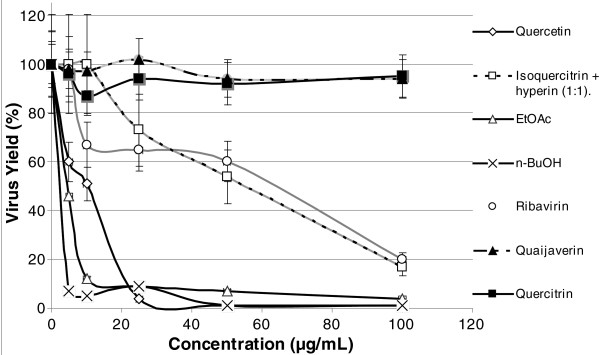
**Anti-MAYV activity of different substances from *B. longifolia*.** The anti-MAYV activity of purified flavonoids or EtOAc and *n*-BuOH extracts from *B. longifolia* was evaluated by treating MAYV-infected cells with 0–100 μg/ml of these substances for 24 h, and then staining for viral plaque counting. The graph shows the results from three independent experiments. Data are presented as mean% virus yield (compared to untreated controls) ± SD.

Quercetin, EtOAc, and *n*-BuOH fractions inhibited MAYV replication in Vero cells in a dose-dependent manner, with more than 90% inhibition at 25 μg/mL (Table 2 and Figure 3). In contrast, mixtures of the isomers isoquercitrin (**4a**) and hyperin (**4b**) or the antiviral ribavirin were much less potent inhibitors of MAYV replication, with IC_90_ values above 100 μg/mL (Table 2 and Figure 3). The monoglycosides guaijaverin and quercitrin did not show significant antiviral activity in the concentrations tested here (Table 2). EtOAc had the highest Selectivity Index (SI), which means that the concentration necessary for antiviral activity is much lower than that causing toxicity to these cells. A high SI is desirable, although much lower values warrant further investigation (see the value for ribavirin obtained under the conditions of this study). The Relative Potency was also calculated using ribavirin as a reference, and n-BuOH was 20 times more powerful than ribavirin in these conditions. EtOAc (4) and quercetin (5) were also more powerful than ribavirin.

**Table 2:** **Cytotoxicity and anti-MAYV activity of purified quercetin and its derivatives EtOAc and *n*-BuOH extracts, containing equal parts of these compounds**

**Substance**	${\mathrm{CC}}_{\mathrm{50}}^{a}$	${\mathrm{CC}}_{\mathrm{90}}^{a}$	${\mathrm{IC}}_{\mathrm{50}}^{b}$	${\mathrm{IC}}_{\mathrm{90}}^{b}$	**SI**^**c**^	**RP**^**d**^
	**μg/ml**	**μg/ml**	**μg/ml**	**μg/ml**		
**Guaijaverin**	795 ± 54	53 ± 5	>100	>100	Nd	nd
**Quercetin**	941 ± 123	43 ± 4	10 ± 0.7	19 ± 0.7	94	5
**Quercitrin**	411 ± 46	41 ± 4	>100	>100	Nd	nd
**Isoquercitrin + hyperin (1:1)**	265 ± 19	135 ± 11	59 ± 0.2	105 ± 3	4	1
**EtOAc**	3116 ± 405	49 ± 5	5 ± 0.3	25 ± 3.5	623	4
***n*-BuOH**	418 ± 47	64 ± 3	3 ± 0.2	5 ± 0.3	208	20
**Ribavirin**	523 ± 42	215 ± 6	62 ± 4	112 ± 8	8	nd

^a^ –50% and 90% cytotoxic concentration.

^b^ –50% and 90% inhibitory concentration of viral replication.

^c^ – Selectivity Index = CC_50_/IC_50_.

^d^ – Relative Potency = standard IC_90_ / substance IC_90_.

nd – Not determined.

## Discussion

In this study, the antiviral activity of quercetin and quercetin derivatives from *B. longifolia* on the replication of the arbovirus Mayaro was investigated. EtOAc and *n*-BuOH fractions containing a mixture of five flavonoids, including quercetin and its glycoside derivatives (guaijaverin, quercitrin, isoquercitrin, and hyperin, in different proportions) displayed stronger antiviral activities than those observed for the purified compounds and for the antiviral ribavirin, used as a positive control. Quercetin and the mixture of isoquercitrin and hyperin in identical proportions showed significant anti-MAYV activity while guaijaverin and quercitrin did not.

Importantly, quercetin displayed better selectivity towards MAYV and improved potency (SI = 94 and RP = 5) when compared to the antiviral ribavirin (SI = 8), clinically approved for the treatment of Hepatitis C virus infection and also experimentally shown to inhibit several other RNA and DNA viruses such as influenza, hepatitis B, poliomyelitis, measles, and smallpox [37]. Also, the antiviral activity of quercetin was considerably stronger and more selective than that of glycosilated quercetin derivatives, suggesting that glycosilation reduces the antiviral activity of quercetin against MAYV. In contrast, Yarmolinsky *et al.*[38] reported increased anti-herpes simplex virus-1 (HSV-1) activity for the compounds rutin (quercetin-3-*O*-rutinoside), kaempferol-3-*O*-rutinoside, and kaempferol-3-*O*-robinobioside compared to that of the respective non-glycosylated compounds (quercetin and kaempferol). The difference in the findings may be explained by the fact that MAYV and HSV-1 are viruses with very different replication strategies.

The mixture of isoquercitrin and hyperin had anti-MAYV activity similar to that displayed by ribavirin, but the Selectivity Index was lower (4). This was due to the higher toxicity of the isoquercitrin/hyperin mixture if compared to ribavirin. However, the Relative Potency for ribavirin and the isoquercitrin/hyperin were similar, suggesting possible synergism between these isomeric flavonoids. The absence of anti-MAYV activity for guaijaverin and quercitrin in the concentrations tested here suggests that 3-*O*-glycosilation with arabinose and rhamnose is particularly detrimental to the antiviral activity against MAYV. This is an interesting finding already under investigation by our group.

According to Gould and Lister [39], flavonoids protect the plant against infection by microorganisms. Therefore, it is not surprising that these compounds are effective *in vitro* against a wide array of viruses, including HSP-1, parainfluenza-3, influenza, and dengue virus type-2 (DENV-2) [40-43], even though the antiviral mechanism for quercetin has not yet been fully elucidated. Zandi *et al.*[42] suggested that quercetin might prevent DENV-2 replication by inhibiting viral RNA polymerase, in a similar mechanism to that described for silymarin, a flavonoid mixture effective against the hepatitis C virus [44]. Inhibition of viral RNA metabolism, through binding to virus RNA, is also the most likely mechanism of action of the flavonol kaempferol against the Japanese encephalitis virus (JEV) [45].

## Conclusions

Our results show that *B. longifolia* is a valuable source of flavonoids with antiviral activity against the arbovirus MAYV. Although quercetin and its 3-*O*-glycosides are fairly common in plants, this is the first report of anti-MAYV activity for these flavonoids. Our data are an important step in the evaluation of natural products as sources of novel drugs to be used in combination therapy, to circumvent drug resistance, or to replace currently used antivirals with unwanted cytotoxic effects.

## Competing interests

The authors declare that they have no competing interests.

## Authors’ contributions

RMK and DF conceived and designed the study. AES collected plant material, performed all phytochemical experiments and wrote the initial draft of the manuscript. KAY and TSS performed cytotoxicity and viral yield inhibition assays. MDFM and RC revised the data and carried out statistical analyses. MRS provided invaluable discussions on the chemical data and antiviral chemistry. All authors read and approved the final manuscript.
